# Deletion of Dual Specificity Phosphatase 1 Does Not Predispose Mice to Increased Spontaneous Osteoarthritis

**DOI:** 10.1371/journal.pone.0142822

**Published:** 2015-11-12

**Authors:** Michael Andrew Pest, Courtney Alice Pest, Melina Rodrigues Bellini, Qingping Feng, Frank Beier

**Affiliations:** 1 Department of Physiology and Pharmacology, Western University, London, ON, Canada; 2 Children’s Health Research Institute, London, ON, Canada; Université de Lyon - Université Jean Monnet, FRANCE

## Abstract

**Background:**

Osteoarthritis (OA) is a degenerative joint disease with poorly understood etiology and pathobiology. Mitogen activated protein kinases (MAPKs) including ERK and p38 play important roles in the mediation of downstream pathways involved in cartilage degenerative processes. Dual specificity phosphatase 1 (DUSP1) dephosphorylates the threonine/serine and tyrosine sites on ERK and p38, causing deactivation of downstream signalling. In this study we examined the role of DUSP1 in spontaneous OA development at 21 months of age using a genetically modified mouse model deficient in *Dusp1* (DUSP1 knockout mouse).

**Results:**

Utilizing histochemical stains of paraffin embedded knee joint sections in DUSP1 knockout and wild type female and male mice, we showed similar structural progression of cartilage degeneration associated with OA at 21 months of age. A semi-quantitative cartilage degeneration scoring system also demonstrated similar scores in the various aspects of the knee joint articular cartilage in DUSP1 knockout and control mice. Examination of overall articular cartilage thickness in the knee joint demonstrated similar results between DUSP1 knockout and wild type mice. Immunostaining for cartilage neoepitopes DIPEN, TEGE and C1,2C was similar in the cartilage lesion sites and chondrocyte pericellular matrix of both experimental groups. Likewise, immunostaining for phosphoERK and MMP13 showed similar intensity and localization between groups. SOX9 immunostaining demonstrated a decreased number of positive cells in DUSP1 knockout mice, with correspondingly decreased staining intensity. Analysis of animal walking patterns (gait) did not show a discernable difference between groups.

**Conclusion:**

Loss of DUSP1 does not cause changes in cartilage degeneration and gait in a mouse model of spontaneous OA at 21 months of age. Altered staining was observed in SOX9 immunostaining which may prove promising for future studies examining the role of DUSPs in cartilage and OA, as well as models of post-traumatic OA.

## Introduction

Osteoarthritis (OA) is a degenerative joint disease which is estimated to afflict at least 10% of the US population over the age of 25 [1]. Symptoms of OA include joint pain and stiffness which can become severe enough to limit activity and ability to work. This results in a substantial loss for both patient quality of life and the economy through missed work hours and direct healthcare costs [2]. Currently, pharmacological interventions only mitigate the symptoms of the disease and do not slow, stop or reverse the underlying joint damage associated with OA, and so further research is needed [2]. While the etiology and pathophysiology of OA is poorly understood, research has shown that contrary to previous beliefs that OA was primarily a disease of ‘wear and tear’, there is a complex set of cellular changes linked to genetic factors and altered biomechanics which occurs in the joint tissues and influences disease initiation and progression [3].

At the tissue level, chondrocytes are the only active cellular component of the cartilage that caps the bone in articular joints such as the knee, elbow and ankle. These cells maintain tissue homeostasis by balancing anabolic buildup and catabolic turnover of surrounding extracellular matrix (ECM) proteins [2]. The ECM forms the vast majority of cartilage tissue and consists of a complex network largely composed of collagen II and sulfated glycosaminoglycan (GAG) containing proteoglycans like aggrecan [2]. Matrix production is largely controlled by the transcription factor SRY (sex determining region Y)-box 9 (SOX9), which also acts as the master regulator of the chondrocyte phenotype [2,4]. Conversely, matrix is catabolized by a number of proteinases produced by chondrocytes including matrix metalloproteinase (MMP) 3 and MMP13, as well as various aggrecanases [2]. An imbalance in ECM turnover, and tissue homeostasis is thought to be one of the primary underlying reasons for cartilage degeneration in OA. This imbalance is largely driven by certain growth factors and inflammatory cytokine signalling [2].

Various growth factors have been implicated in OA, including transforming growth factor beta (TGFβ), fibroblast growth factors (FGF), and transforming growth factor alpha (TGFα) [5–7]. Previous studies in our lab have shown TGFα mediated activation of epidermal growth factor receptor (EGFR) induces cartilage degeneration *in vitro*, and inhibition of EGFR *in vivo* partially protects against surgically induced osteoarthritis in rats [8,9]. A portion of the cellular response to TGFα and other EGFR ligands is mediated by activation of downstream mitogen activated protein kinases (MAPKs) such as extracellular signal-regulated kinase 1 and 2 (ERK1/2) and p38 MAPK [8,10–12]. ERK activation has been shown to induce downstream targets involved in OA including MMPs, and blockade of MEK/ERK signalling attenuated cartilage degeneration using *in vitro* cartilage explant models, and *in vivo* rabbit models of OA [8,12,13]. Inhibition of p38 has also been shown to partially attenuate production of catabolic MMPs, and reduce cartilage degeneration and pain-like behaviours in a rat monoiodoacetate (MIA) induced OA model [14,15]. Furthermore, p38 mediates part of the activity of various inflammatory cytokines including IL-1β and TNFα which are important in both inflammatory arthritis and osteoarthritis [2,16].

Dual-specificity phosphatase 1 (DUSP1), also known as MAPK phosphatase-1 (MKP-1), and other DUSP proteins are negative regulators of MAPK signaling [17]. With the ability to dephosphorylate phosphoserine/phosphothreonine and phosphotyrosine sites, DUSP1 can deactivate phosphorylated ERK and p38 [17,18]. DUSP1 has been shown to attenuate MAPK signaling, effectively reducing osteolysis and cartilage degeneration in studies utilizing mouse models of lipopolysaccharide (LPS) induced inflammatory bone loss and collagen induced arthritis (CIA) [16,19,20]. Inhibition of DUSP1 also increased chondrocyte apoptosis, which was exacerbated by the cytokine TNFα in cell culture [21]. Our own studies have shown induction of *Dusp1* expression in chondrocytes by pharmacological inhibition of phosphatidylinositol-3-kinase (PI3K) signaling [22]. However, the role of DUSP1 in OA has not been closely examined using *in vivo* animal models.

In this study, we investigated the role of DUSP1 in spontaneously occurring OA by aging mice genetically deficient in *Dusp1*. We showed that both female and male mutant animals show similar disease progression to controls at 21 months of age.

## Methods

### Animals

All animals were bred in-house, raised, and sacrificed in accordance to the ethical guidelines of the Canadian Council on Animal Care (CCAC). Animal use protocols were approved by the Council on Animal Care at Western University—Canada (Animal Use Permit: 2015–031). Mice were housed on a 12 hour light/dark cycle in standard shoebox caging with free access to mouse chow and water, but without access to running wheels or other exercise based enrichments. Mice with a whole body deletion for *Dusp1* were obtained from QF on a mixed C57Bl/6 and 129S2 background [23,24]. Trials were setup in cages containing at least 1 pair of littermate matched wild type and knockout animals, with an average of 3–4 mice per cage for the duration of the experiment. All animals were weighed prior to euthanization by asphyxiation with CO_2_. Wild type (*Dusp1* ^+/+^, WT), heterozygous (*Dusp* ^*+/-*^, Het), and DUSP1 knockout (*Dusp1* ^-/-^, KO) mice were genotyped using standard PCR techniques on ear tissue biopsies using the following primers:

*Dusp1* For1–5-CCA GGT ACT GTG TCG GTG GTG C-3, *Dusp1* For2–5-TGC CTG CTC TTT ACT GAA GGC TC-3, *Dusp1* Rev1–5-CCT GGC ACA ATC CTC CTA GAC-3 [25].

### Histology

Limbs were collected and fixed in 4% paraformaldehyde for 24 hours. The intact joints were then decalcified in 5% ethylenediaminetetraacetic acid (EDTA) in phosphate buffered saline (PBS, pH 7.0) for 10 to 12 days at room temperature. Knee joints were processed and embedded in paraffin to obtain sections 5 μm thick in the frontal orientation. Staining with toluidine blue for loss of glycosaminoglycans (GAGs) and cartilage structural changes was conducted as previously described [26].

### Articular Cartilage Evaluation

To evaluate OA related joint degeneration, 5 evenly spaced sections spanning a region approximately 500 μm were examined. OA related damage was assessed using the OARSI approved histological scoring system for mice [27], with all four quadrants of the joint (medial/lateral tibia and femur) scored by at least 2 blinded observers. Cartilage damage scores are reported as the summed OARSI score of the 5 evaluated slides for each individual quadrant and the total joint (sum of the four quadrants). As previously described, the average articular cartilage thickness was measured from the subchondral bone to the surface of the intact articular cartilage at three evenly spaced points along the width of each knee joint quadrant using frontally oriented sections [26]. Sections from 5 slides spanning approximately 500 μm were evaluated, and measures were averaged over the width and depth of each quadrant to give the average articular cartilage thickness.

### Immunohistochemistry

Primary antibodies for cartilage and bone matrix breakdown products were received as a gift from Dr. John Mort and included anti-TEGE (aggrecanase mediated aggrecan cleavage neoepitope), anti-DIPEN (matrix metalloproteinase mediated aggrecan cleavage neoepitope) and anti-C1,2C (a collagen I and II cleavage neoepitope) and were utilized as previously described [28–31]. Additional antibodies were obtained from their various manufacturers: MMP13 (Proteintech), SOX9 (R&D Systems), phosphoERK1/2 (Cell Signalling). Sections were incubated for 15 minutes in 3% H_2_O_2_ in methanol to eliminate endogenous peroxidase activity, followed by blocking in 5% goat or donkey serum in PBS, and overnight incubation with primary antibody at 4°C or with no primary antibody for use as a control. Additional controls were performed with rabbit or goat normal IgG (Santa Cruz) under the same conditions (S3 Fig). Sections were incubated with the appropriate secondary antibody (goat anti-rabbit, or donkey anti-goat, Santa Cruz) conjugated to horseradish peroxidase (HRP) and developed using DAB+ chromogen (Dako Canada). Sections were counterstained with methyl green, dehydrated in solutions of increasing ethanol concentration ending in xylene, and cover slipped.

### CatWalk Gait Analysis

Changes in gait and weight bearing were assessed using the Noldus CatWalk system [32–34]. Animals were transferred to a behavioral facility 2 days prior to assessment. In brief, animals were allowed to walk freely through a semi-enclosed tunnel along an internally illuminated glass plate. In a dark room the scattering of the light caused by interaction with the animal’s paws is detected by a camera, and subsequently interpreted by the accompanying software (Noldus CatWalk v7.1) as various gait parameters. Stride length is calculated as the average distance between paw strikes for each limb. Duty cycle is an expression of the percentage of time the paw remains in contact with the glass plate, relative to the total gate cycle as shown in this equation: Duty Cycle = [Stand Time/(Stand Time + Swing Time)] x 100%. Print area and paw intensity are related to the amount of weight the animal bears on an individual paw, with the maximal contact area and maximal intensity of light measured respectively.

### Statistical Analysis

All data was analyzed for statistical significance using GraphPad Prism (v6.0) and the appropriate statistical techniques (Kruskal-Wallis test with Dunn’s Correction or Mann-Whitney test as indicated). Outliers were assessed using the Grubbs’ outlier test.

## Results

### DUSP1 KO animals show good general health up to 21 months of age

At 21 months of age, most DUSP1 KO, Het, and WT control mice appeared to be generally healthy. A small number of animals either demonstrated repeated skin ulcerations and were withdrawn from the trial, or died from unknown causes prior to 21 months of age (S1 Table). Animal weights were taken at 21 months immediately prior to sacrifice to evaluate possible metabolic changes due to loss of DUSP1. Female animals showed no statistically significant difference in weight, however male WT mice were on average 7.8 g heavier (p = 0.038) than their KO littermates (S1 Fig).

### DUSP1 KO animals are not protected from the histological changes associated with spontaneous OA

**Fig 1:**
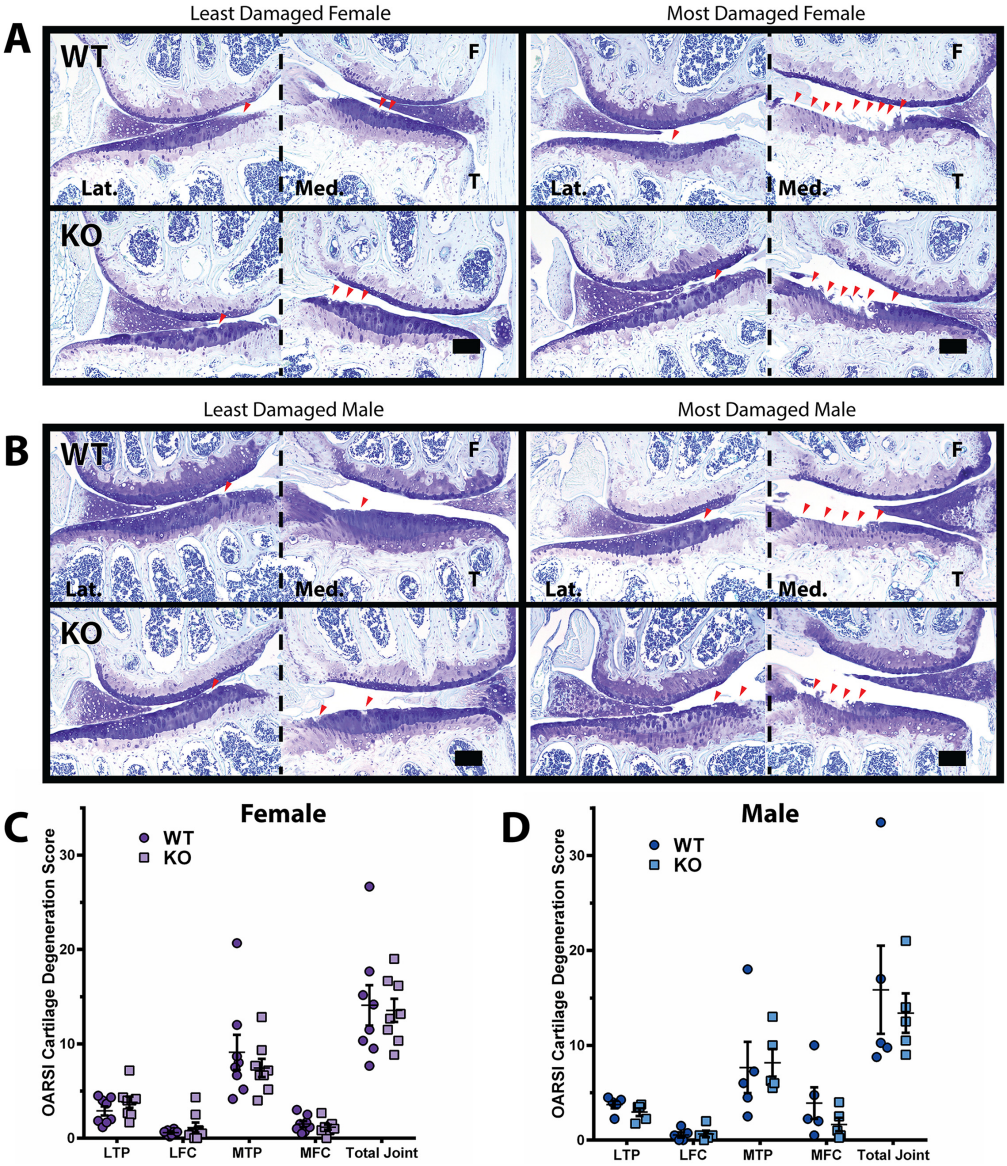
21 month old *Dusp1* KO mice show similar cartilage damage as controls. DUSP1 KO and WT control animals were aged to 21 months and right knees were evaluated for cartilage damage following OARSI recommended guidelines. **(A-B)** Representative toluidine blue stained frontal knee section images of the lateral (Lat.) and medial (Med.) femur (F) and tibia (T) of **(A)** female (n = 8) and **(B)** male (n = 5) mice, least damaged (left) and most damage (right), wild type controls (WT, top) and DUSP1 knockout (KO, bottom). Lesion (red arrowheads) location patterns and size were similar between WT and KO groups. Scale bars = 100 μm. **(C-D)** Total OARSI cartilage degeneration scores across the lateral tibial plateau (LTP), lateral femoral condyle (LFC), medial tibial plateau (MTP), medial femoral condyle (MFC), and cumulative joint score (Total Joint) of **(C)** female (n = 8) and **(D)** male (n = 5) WT and KO mice. OARSI scores did not show statistically significant differences between WT and KO groups when analyzed by Kruskal-Wallis with Dunn's multiple comparison test. Error bars are shown as mean ± SEM.

At 21 months of age, DUSP1 KO and WT littermate control mice were sacrificed and knee joints harvested for histological evaluation of joint health. Slides of frontally embedded, toluidine blue stained mouse knees were evaluated for OA related histological changes by at least 2 blinded observers, using the OARSI recommended cartilage degeneration scoring system [27]. In both female (n = 8) and male (n = 5) trials of DUSP1 KO and WT control mice, no statistically significant differences in joint degeneration were observed in the individually examined joint quadrants, nor the summed total joint score (Fig 1A–1D and S2 Table). Similarly, when we compared the scores of male to female cohorts, no significantly different findings were observed between any pairing of groups. In both male and female cohorts, a single animal showed larger than average cartilage degeneration, particularly in the medial tibial plateau (MTP). When these points were assessed as outliers using the Grubbs’ test and excluded, the results noted did not change conclusions on statistical significance, so they remained included for the reported data (Fig 1C and 1D).

**Fig 2:**
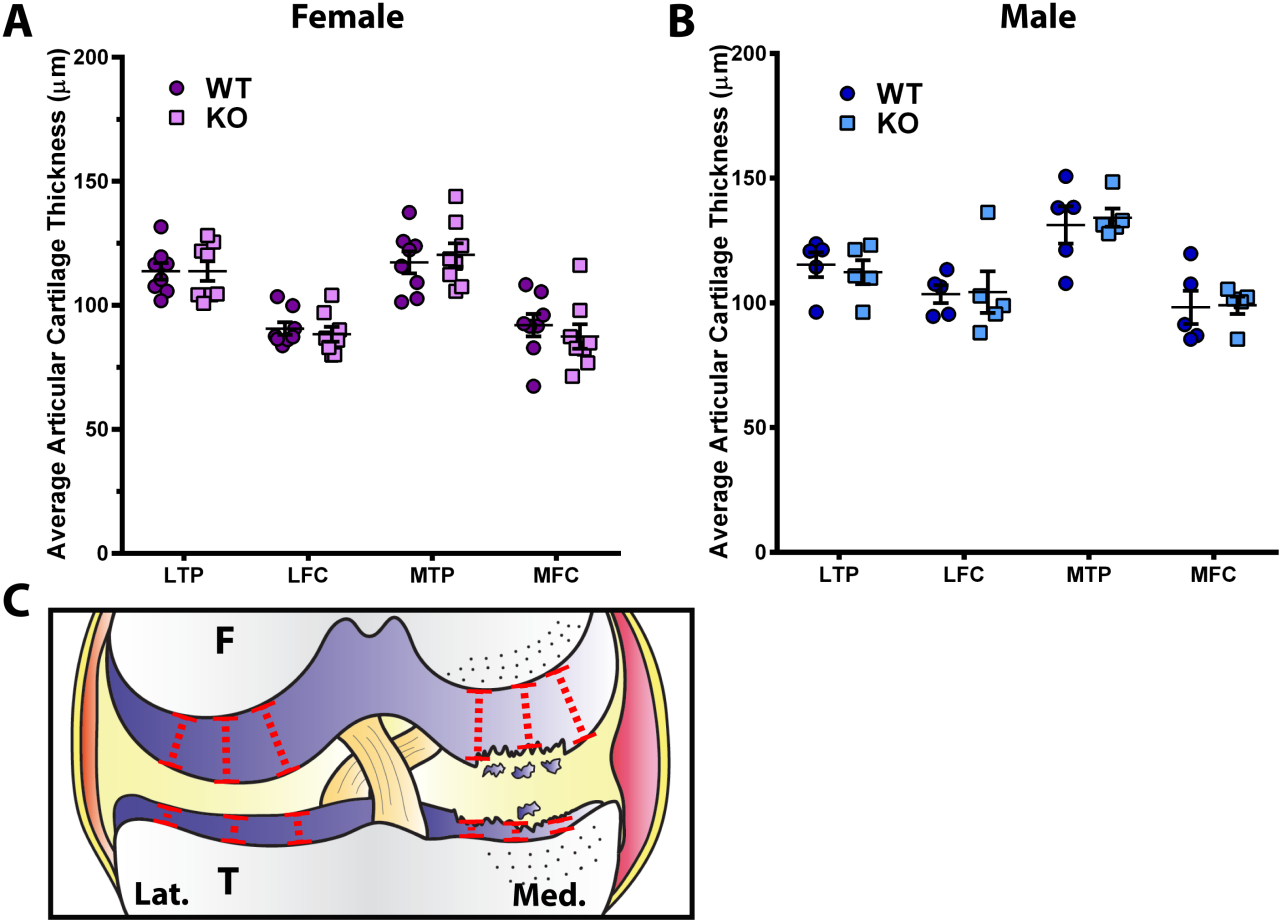
*Dusp1* KO mice show similar articular cartilage thickness when compared to controls. Measurements from the subchondral bone to the intact articular cartilage surface were made at 3 points across the lateral tibial plateau (LTP), lateral femoral condyle (LFC), medial tibial plateau (MTP) or medial femoral condyle (MFC) and averaged. **(A)** Female (n = 8) and **(B)** male (n = 5) DUSP1 KO mice do not show statistically significantly different articular cartilage thickness when compared to WT controls. Analyzed by Kruskal-Wallis with Dunn's multiple comparison test. Error bars are shown as mean ± SEM. **(C)** Example diagram demonstrating approximate sites of articular cartilage measurements indicated by dashed red lines. F = femur, T = tibia, Lat. = lateral, Med. = medial.

To directly evaluate any differences in the articular cartilage thickness in the knee joints of DUSP1 KO and control WT mice, the distance from the subchondral bone to the surface of the intact articular cartilage was measured (Fig 2C). No statistically significant differences were observed when comparing male KO to WT or female KO to WT animals (Fig 2A and 2B and S3 Table).

### DUSP1 KO mice show similar levels of cartilage breakdown products

Following the catabolic activities of various metalloproteinases (MMPs, aggrecanases, etc.) on ECM proteins, neoepitopes are formed at either side of the cleavage site, and can be detected using specialized antibodies. We utilized antibodies for neoepitopes formed through aggrecan and collagen II cleavage to evaluate any differences in the pattern of cartilage degeneration in our DUSP1 KO mice. The DIPEN neoepitope is formed when the interglobular domain (IGD) of the aggrecan core protein is cleaved by MMPs, and represents the new N-terminal of the cleaved aggrecan protein [29]. Likewise, the TEGE neoepitope represents the new N-terminal in the IGD of aggrecanase cleaved aggrecan [30]. Collagen breakdown product neoepitopes can also be similarly identified. The products of MMP mediated cleavage of both collagen I and II form ¾ and ¼ length fragments. The C-terminal of the ¾ fragment is known as the neoepitope C1,2C [31].

**Fig 3:**
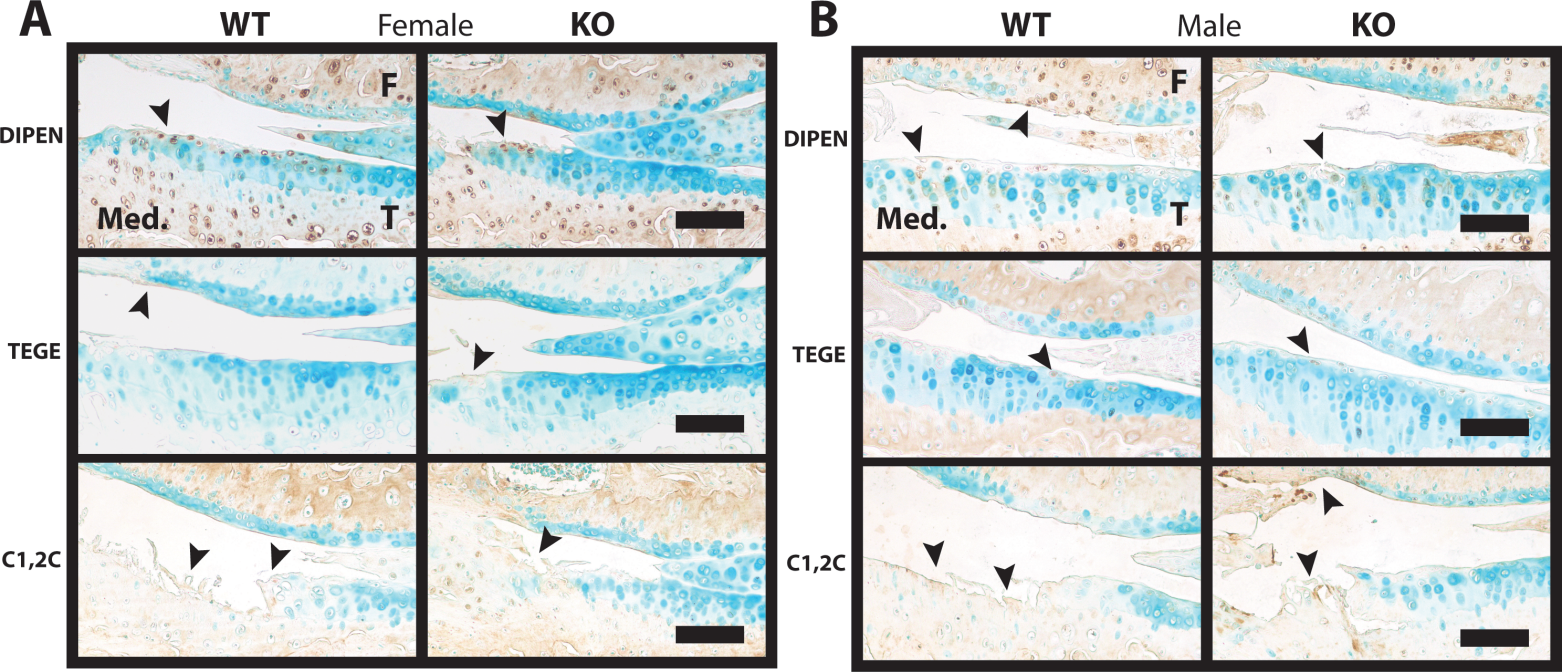
Cartilage matrix neoepitope immunostaining shows similar intensity and localization in *Dusp1* KO and WT control mice. **(A)** Female (n = 6) and **(B)** male (n = 5) representative medial femur (F) and tibia (T) joint sections were immunostained for: DIPEN (Top, MMP cleaved aggrecan neoepitope), TEGE (Middle, aggrecanase cleaved aggrecan neoepitope), and C1,2C (Bottom, MMP cleaved collagen II/I). Regions of pericellular and extracellular matrix (ECM) staining are indicated by the black arrowheads. Scale bars = 100 μm.

No large qualitative differences could be identified when examining immunostained knee sections from either female (n = 6, Fig 3A) or male (n = 5, Fig 3B) DUSP1 KO and WT control mice. Staining for DIPEN was most pronounced in the matrix of the cartilage lesion, with additional chondrocyte pericellular matrix staining observed in many animals (Fig 3). Weak matrix staining for the TEGE neoepitope was observed in the ECM of cartilage lesions, however little chondrocyte pericellular matrix staining was evident in the MTP (Fig 3). Conversely, increased staining was evident in the pericellular region of chondrocytes in the lateral tibial plateau (LTP) and lateral femoral condyle (LFC) of both male and female animals (data not shown). C1,2C immunostaining was found present only in ECM of the cartilage lesion sites (Fig 3).

Interestingly, the growth plates (tibia and femur) of both female and male animals showed intense DIPEN immunostaining throughout the cartilage matrix (S2 Fig), but no differences between genotypes were observed. Immunostaining for MMP13 in the same regions did not appear to be particularly strong, and no differences were noted between WT and KO for either female or male groups (S2 Fig). Both normal IgG and no primary antibody controls did not show any abnormal background staining in cartilage (S3 Fig).

### Loss of DUSP1 causes an imbalance in anabolic but not catabolic chondrocyte activity

**Fig 4:**
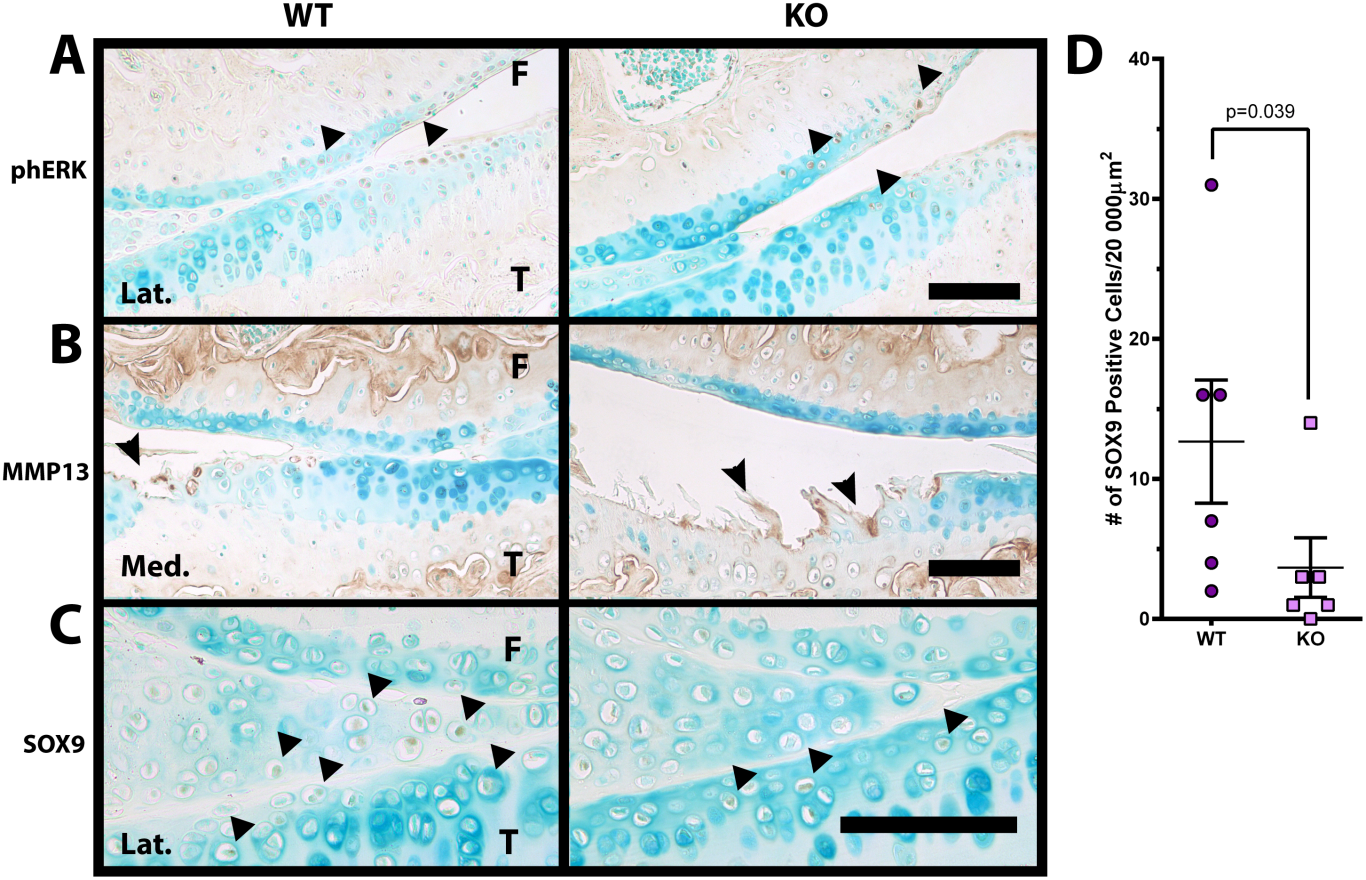
Decrease in cartilage anabolism marker SOX9 in *Dusp1* KO mice. **(A)** Representative phosphoERK (phERK) immunostained female (n = 6) lateral (Lat.) femur (F) and tibia (T) joint images show similar cellular staining (arrowheads) in DUSP1 KO and WT control mice. **(B)** Representative MMP13 immunostained female (n = 6) medial (Med.) femur (F) and tibia (T) joint images show similar pericellular and matrix staining (arrowheads) in DUSP1 KO and WT control mice. **(C)** Representative SOX9 immunostained female (n = 6) medial femur (F) and tibia (T) joint images show decreased staining intensity and positive cells (arrowheads) in *Dusp1* KO mice when compared to WT controls. All scale bars = 100 μm. **(D)** The number of SOX9 positive cells in female *Dusp1* KO mice was decreased but not statistically significantly different (p = 0.095) from WT controls. Data analyzed using Mann-Whitney test. Error bars are shown as mean ± SEM.

The DUSP1 target ERK mediates the effects of many cell surface receptors including EGFR. Immunostaining for the phosphorylated activated form of ERK (phERK) showed similar staining patterns and intensity in the lateral knee compartment of female DUSP1 KO and WT animals (Fig 4A) and males (data not shown). However, little to no cellular staining was observed in the medial compartment of the knee for any group (data not shown).

Matrix metalloproteinase 13 (MMP13) cleaves both collagen II (C1,2C neoepitope) as well as aggrecan ECM molecules (DIPEN neoepitope), and has been implicated as one of the key mediators of cartilage destruction in OA [35]. Immunostaining for MMP13 in knee joint articular cartilage was localized largely to the chondrocyte pericellular ECM and surrounding cartilage matrix immediately adjacent to lesion sites in female (Fig 4B) and male (data not shown) mice. Little difference was noted for MMP13 staining between WT and KO animals, which is compatible with previous observations for DIPEN and C1,2C neoepitope staining as noted above.

SOX9 is the master regulator of the chondrocyte cellular phenotype, and is important in both establishing a chondrocyte lineage during development as well as maintaining that phenotype in adult tissues [4]. In the lateral compartment of the knee joints of female DUSP1 KO mice the intensity of SOX9 immunostaining was decreased in the articular cartilage chondrocytes and meniscus when compared to WT controls (Fig 4C). Similar results were observed in male animals (data not shown), however, little cellular staining was observed in the medial compartment of either sex (data not shown). Similarly, the number of SOX9 positive cells counted in a 200 x 100 μm box set at the surface of the lateral tibial plateau in female animals demonstrated a decrease in KO when compared to WT mice (p = 0.039) (Fig 4D). Similar findings were noted when the number of SOX9 positive cells was normalized to animal weight to correct for potential metabolic or loading effects (S4 Fig).

### The gait of DUSP1 KO mice is comparable to WT mice

**Fig 5:**
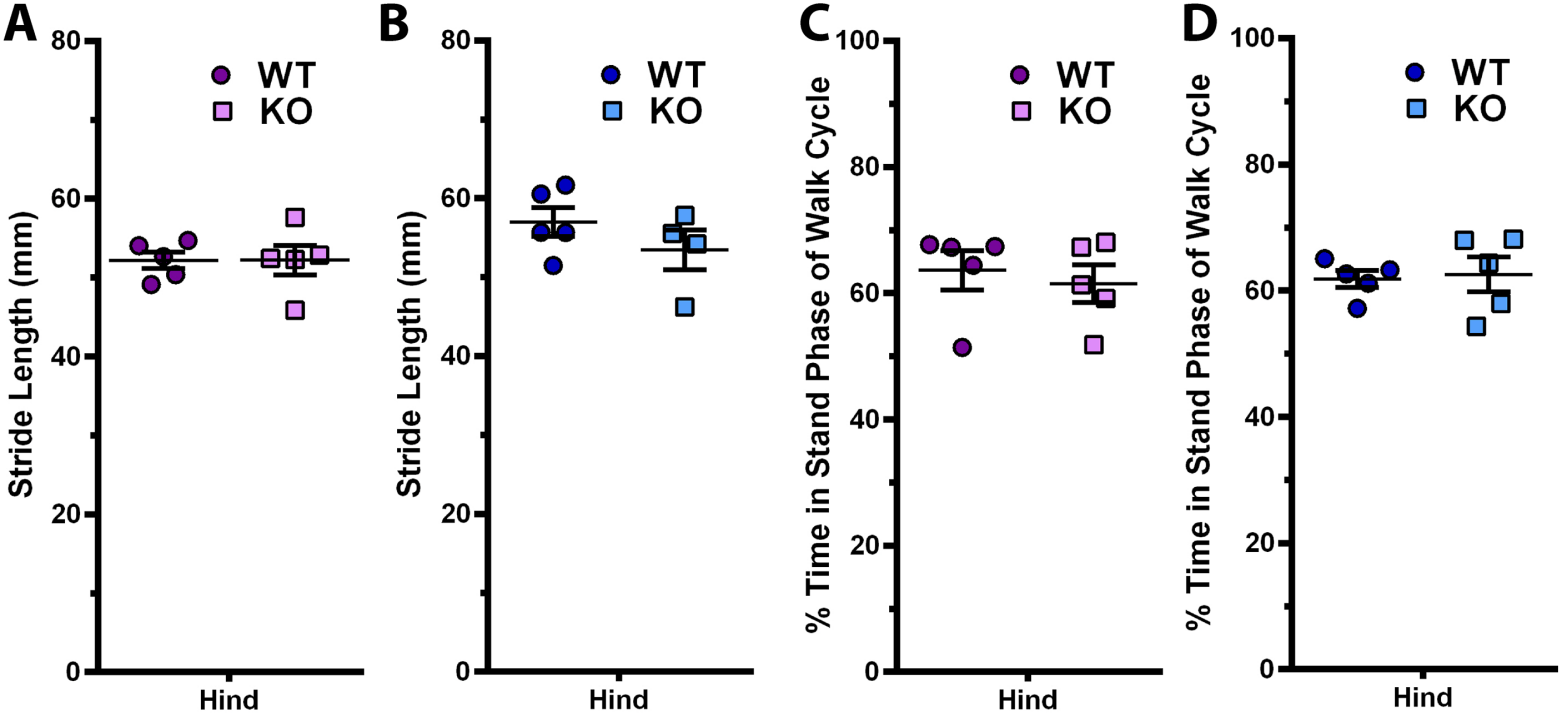
Gait patterns are not different in *Dusp1* KO mice at 21 months of age. Gait patterns of *Dusp1* KO and WT control mice were measured using the Noldus CatWalk apparatus prior to sacrifice at 21 months of age. **(A)** Female (n = 5) and **(B)** male (n = 5) stride length (distance between individual hind paw prints during gait) were not statistically different between WT and KO mice. **(C)** Female (n = 5) and **(D)** male (n = 5) duty cycle (% of time in stand phase of walk cycle) was also not statistically different between WT and KO mice. Data analyzed using Mann-Whitney test. Error bars are shown as mean ± SEM.

To evaluate changes in the gait patterns of our aged DUSP1 KO mice vs WT controls, we utilized the Noldus CatWalk apparatus, which measures parameters of rodent walking patterns. We examined 4 of these parameters, including stride length, duty cycle, print area and paw intensity. No statistically significant differences were found comparing the stride length of the hind limbs of WT vs KO female (Fig 5A) and male (Fig 5B) animals. Similarly, duty cycle (% time in stand phase of walk cycle) was not found to be statistically significantly different between WT vs KO female (Fig 5C) and male (Fig 5D) animals. For both print area and paw intensity, no statistically significant differences were observed between female or male WT vs KO animals when normalized to animal weights (data not shown).

## Discussion

In this study on the effects of loss of DUSP1 on spontaneous OA in mice, we demonstrated little difference between both female and male DUSP1 KO and WT control mice in terms of OA related cartilage damage and gait changes.

Spontaneous OA can be particularly difficult to study as it may take months to years to develop even in rodent models which develop spontaneous OA-like cartilage damage within 2 years of life [36]. This is in contrast to induced OA models using surgical or chemical methods which model more rapidly developing post-traumatic OA in months or less [36]. Our lab has previously examined mice on the C57Bl/6 background at 21 months of age and has found that they develop mild to moderate OA by this time point (unpublished data). Other studies have shown similar results [37]. While our lab has also shown that F1 C57Bl/6 x 129/SV crossed mice develop mild to moderate OA by 2 years of age [38], future studies should consider using sufficiently backcrossed congenic C57Bl/6 or 129 strain mice to reduce possible confounding genetic variability. However, the severity of OA observed on our mixed background was similar to that seen in our previous work in C57Bl/6 mice.

Previous studies indicate the importance of both ERK and p38 in the progression and initiation of arthritis [8,15] as well as the role of DUSP1 in the negative regulation of these MAPKs [17,24,39]. Despite this we were unable to show any difference in cartilage degeneration when *Dusp1* was genetically removed from our mouse model. This may be in part due to the incredibly complex and dynamic regulation of these particular factors. Both ERK and p38 are regulated at multiple levels (expression, post-translational modification, etc.), with various negative and positive feedback loops, and many different signalling pathways converge on these molecules [10,40]. Therefore, while we have removed DUSP1 from our KO mice, the net effect may have been largely mitigated by increased negative feedback or various other factors. Furthermore, other DUSP protein family members (Reviewed in [17]) may have also compensated for loss of DUSP1 in our model, which is consistent with the minimal changes in cartilage phERK immunostaining observed in KO animals (Fig 4A). Future studies may need to focus further upstream at the receptor level, or downstream at specific transcription factors to gain more insight into the pathobiology of OA and related diseases.

Interestingly, in this study we demonstrated a large increase in the immunostaining for MMP cleaved aggrecan neoepitope DIPEN in the growth plate of our aged animals. To our knowledge, this has not been previously described. Oddly, MMP13 which is considered one of the primary proteinases in cartilage ECM breakdown [2,35] did not show particularly robust staining in the same regions. This may be due to the time point at which we examined our animals, particularly if MMP13 is transiently expressed and the cleaved aggrecan fragments are unable to diffuse out of the growth plate due to otherwise intact ECM protein network structure or restriction by surrounding bone.

In this study we demonstrated a small but statistically significant decrease in SOX9 staining with fewer stained cells found in the tibial plateau of DUSP1 KO animals (Fig 4C and 4D). Previous studies have shown a negative regulatory role for ERK in SOX9 driven chondrogenesis [41,42]. Furthermore, studies in our lab have shown that MEK/ERK inhibition can reverse TGFα/EGFR mediated repression of *Sox9* expression [8]. However, regulation of SOX9 is complex and poorly understood, and other studies have shown that ERK increases SOX9 at the mRNA and protein levels [26,43,44], and p38 may stabilize Sox9 mRNA [45]. However, since our study did not show a large difference in phERK immunostaining, temporal factors, and other DUSP proteins or unknown pathways may be involved in compensating for loss of DUSP1 *in vivo*. Further research is required to elaborate on the role of DUSP1 and MAPK signalling in the regulation of SOX9.

While the weight of the male DUSP1 control animals was on average higher than the KO group, we did not observe any differences in OA development which is known to be influenced by obesity in humans [2]. It is unknown why the female cohort did not demonstrate a similar difference in weights, but this may be due to differences in hormonal control in males and females which is influenced by ERK signalling and may have been modulated by whole body loss of DUSP1 in this study [46].

While evaluating limb pain in rodent models is difficult to assess and widely debated, analysis of gait changes has begun to gain favour as a measure of behaviours related to OA-like pain and joint dysfunction [33,34,47]. To evaluate gait changes possibly due to OA development in our study, we utilized the Noldus CatWalk gait analysis system. While we were unable to show any differences between DUSP1 KO and control mice in the gait variables examined, it is still possible that these animals experienced increased OA related changes in gait. Due to the limitations of the equipment, and the nature of spontaneous OA presenting as a bilateral or unilateral disease, any small but present changes in gait pattern may have been lost in the experimental noise. Interestingly, mice have been shown to mask signs of pain with male observers (MP performed all CatWalk data acquisition), possibly making it difficult to assess changes in gait and perhaps OA related pain-like behaviours [48]. Furthermore, it is difficult to determine the cause of gait changes, as muscle weakness due to aging, physical dysfunction of the joint due to cartilage or bone degeneration, and pain all may influence animal gait behaviours.

In conclusion, we were unable to demonstrate any definitive evidence that DUSP1 plays an important role in spontaneous OA development in mice. However, future research is needed to evaluate if DUSP1 may play a role in the more rapid progression of cartilage degeneration as seen in post-traumatic OA, and if other DUSP proteins can compensate for the loss of DUSP1.

## Supporting Information

S1 Fig Male *Dusp1* KO are lighter than WT controls. Animal weights were taken at 21 months prior to sacrifice. Male (n = 5) *Dusp1* KO mice were on average 7.8 grams lighter than WT controls. Female (n = 6) *Dusp1* KO and WT mice showed no statistically significant differences in weight. Data analyzed by two-way ANOVA with Bonferroni’s multiple comparisons test. Error bars are shown as mean ± SEM.
(TIF)

S2 Fig Intense DIPEN but not MMP13 immunostaining in the growth plate of *Dusp1* KO and WT mice. Female frontal knee sections were immunostained for **(A)** DIPEN (MMP cleaved aggrecan neoepitope) and **(B)** MMP13. DIPEN staining in both WT and KO growth plates is intense despite poor staining for MMP13. Scale bar = 100 um. Representative images shown. N = 6.
(TIF)

S3 Fig Immunostaining controls show little confounding background staining. Female frontal knee sections were immunostained for **(A)** cartilage matrix neoepitopes DIPEN [with growth plate (**GP**)], TEGE, and C1,2C with appropriate rabbit normal IgG control and no primary added controls. **(B)** Immunostaining for MMP13 and phosphorylated ERK (phERK) with rabbit normal IgG control and no primary added controls. **(C)** Immunostaining for SOX9 with goat normal IgG and no primary added controls. Scale bars = 100 μm. Representative images shown. N≥3.
(TIF)

S4 Fig *Dusp1* KO mice show decreased SOX9 positive cells in immunostained knee joint sections when normalized to animal weight. The number of SOX9 positive cells within a 200 x 100 μm box set at the articular cartilage surface of the lateral tibial plateau were counted and normalized to the animal’s weight to correct for any variability caused by differences in joint loading and animal size. *Dusp1* KO mice show decreased numbers of SOX9 positive cells relative to WT controls. Data is presented as individual data points with mean ± SEM. Data analyzed using Mann-Whitney test.
(TIF)

S1 Table *Dusp1* KO mice are generally healthy at 21 months. DUSP1 WT (*Dusp1* ^*-/-*^ *)*, Het (*Dusp1* ^*+/-*^), KO (*Dusp1* ^*-/-*^) animals live and healthy (**Live**) at the end of the experiment, or removed from the experiment due to recurrent skin ulcerations/died of unknown causes (**Dead/Rem**.).
(DOCX)

S2 Table Knee cartilage damage score descriptive statistics. Articular cartilage damage was evaluated using the OARSI recommended scoring system. SEM, standard error of the mean; WT, wild type; KO, knockout; LTP, lateral tibial plateau; LFC, lateral femoral condyle; MTP, medial tibial plateau; MFC, medial femoral condyle.
(DOCX)

S3 Table Knee cartilage thickness descriptive statistics. Articular cartilage thickness was measured from the cartilage surface to the subchondral bone. SEM, standard error of the mean; μm, micrometers; WT, wild type; KO, knockout; LTP, lateral tibial plateau; LFC, lateral femoral condyle; MTP, medial tibial plateau; MFC, medial femoral condyle.
(DOCX)

## Abbreviations

CIA: Collagen induced arthritis
DUSP1: Dual specificity phosphatase 1
ECM: Extracellular matrix
EDTA: ethylene diaminetetraacetic acid
EGFR: Epidermal growth factor receptor
ERK: Extracellular signal-regulated kinase
FGF: Fibroblast growth factor
GAG: Glycosaminoglycan
IL-1β: Interleukin 1 beta
KO: Knock out
LFC: Lateral femoral condyle
LPS: Lipopolysaccharides
LTP: Lateral tibial plateau
MAPK: Mitogen activated protein kinase
MFC: Medial femoral condyle
MIA: Monoiodoacetate
MKP1: MAP (mitogen activated protein) kinase phosphatase 1
MMP: Matrix metalloproteinase
MTP: Medial tibial plateau
OA: Osteoarthritis
p38: p38 mitogen activated protein kinase
PBS: phosphate buffered saline
PI3K: Phosphatidylinositol-3-kinase
SOX9: SRY (sex determining region Y)-box 9
TGFα: Transforming growth factor alpha
TGFβ: Transforming growth factor beta
TNFα: Tumour necrosis factor alpha
WT: Wild type
