# Supplementary figures and images for: Deletion of Dual Specificity Phosphatase 1 Does Not Predispose Mice to Increased Spontaneous Osteoarthritis
Source: PLoS One. 2015 Nov 12;10(11):e0142822. doi: 10.1371/journal.pone.0142822 (PMC4643037; doi:10.1371/journal.pone.0142822)

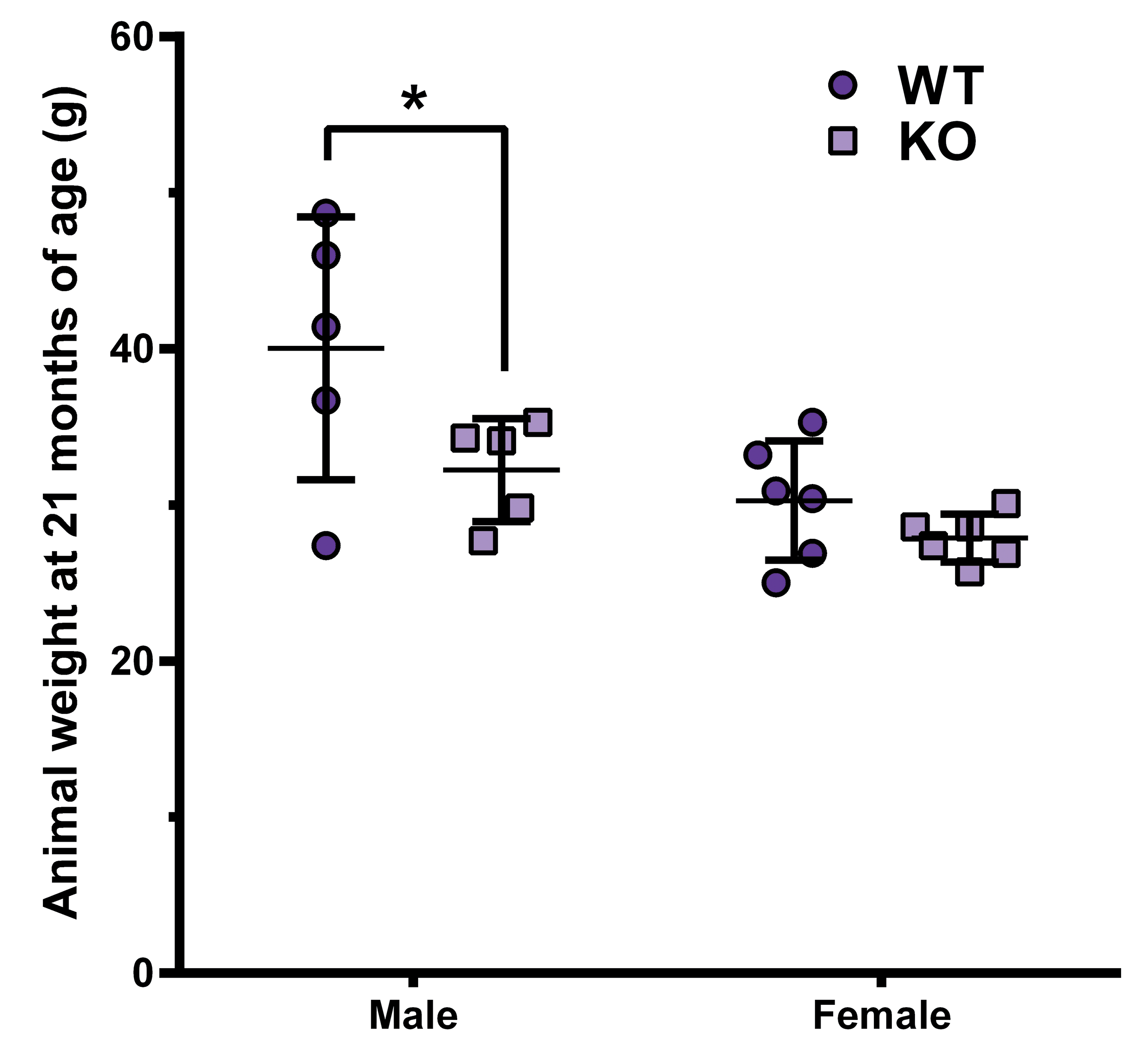

Supplement: S1 Fig — Animal weights were taken at 21 months prior to sacrifice. Male (n = 5) Dusp1 KO mice were on average 7.8 grams lighter than WT controls. Female (n = 6) Dusp1 KO and WT mice showed no statistically significant differences in weight. Data analyzed by two-way ANOVA with Bonferroni’s multiple comparisons test. Error bars are shown as mean ± SEM. (TIF) [file pone.0142822.s001.tif]

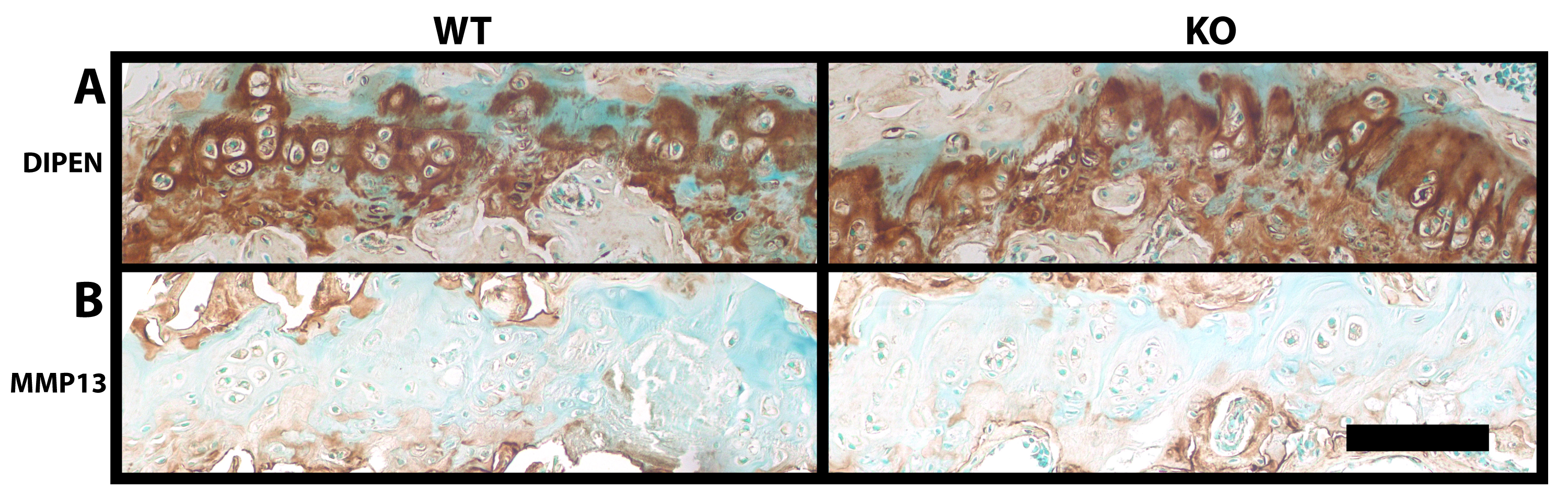

Supplement: S2 Fig — Female frontal knee sections were immunostained for (A) DIPEN (MMP cleaved aggrecan neoepitope) and (B) MMP13. DIPEN staining in both WT and KO growth plates is intense despite poor staining for MMP13. Scale bar = 100 um. Representative images shown. N = 6. (TIF) [file pone.0142822.s002.tif]

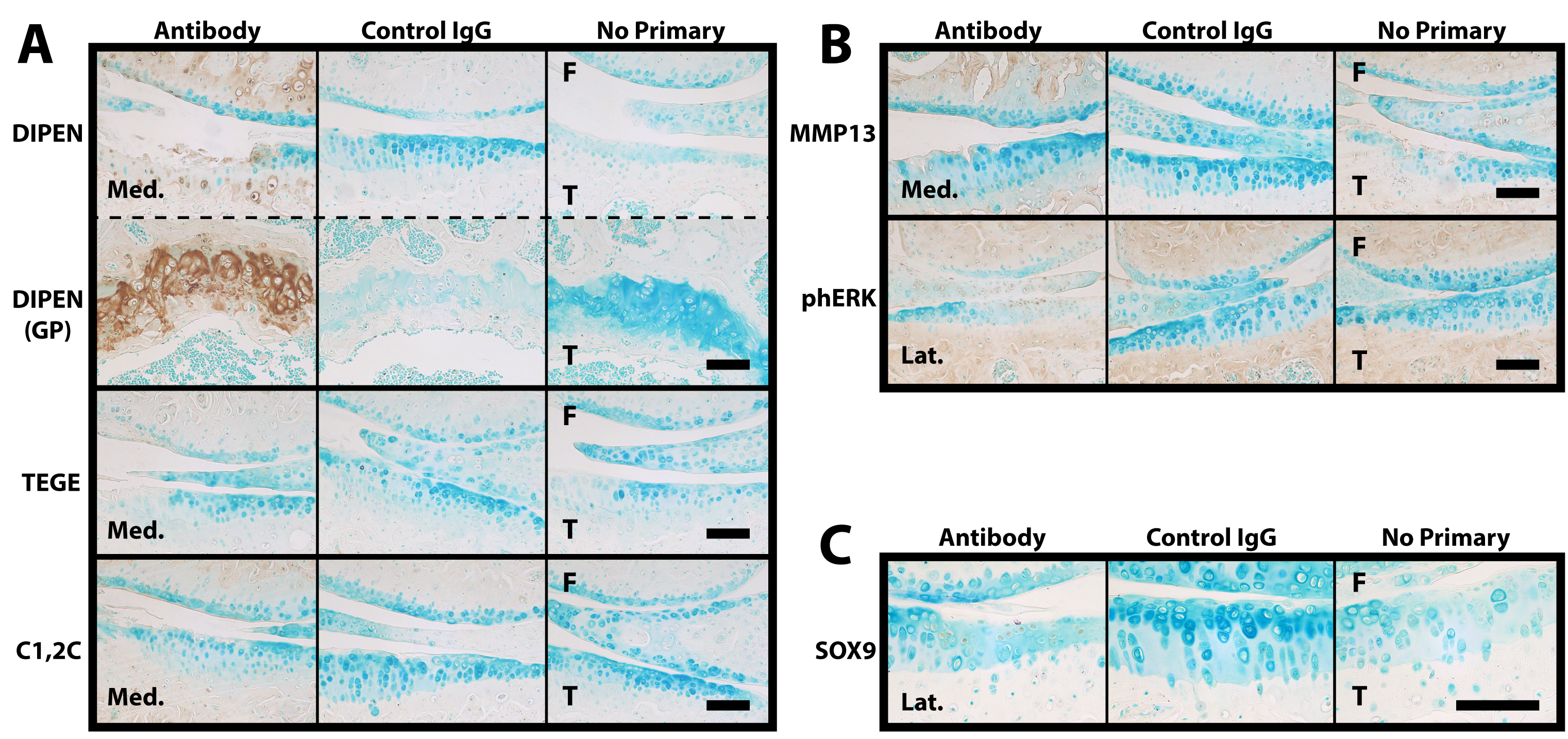

Supplement: S3 Fig — Female frontal knee sections were immunostained for (A) cartilage matrix neoepitopes DIPEN [with growth plate (GP)], TEGE, and C1,2C with appropriate rabbit normal IgG control and no primary added controls. (B) Immunostaining for MMP13 and phosphorylated ERK (phERK) with rabbit normal IgG control and no primary added controls. (C) Immunostaining for SOX9 with goat normal IgG and no primary added controls. Scale bars = 100 μm. Representative images shown. N≥3. (TIF) [file pone.0142822.s003.tif]

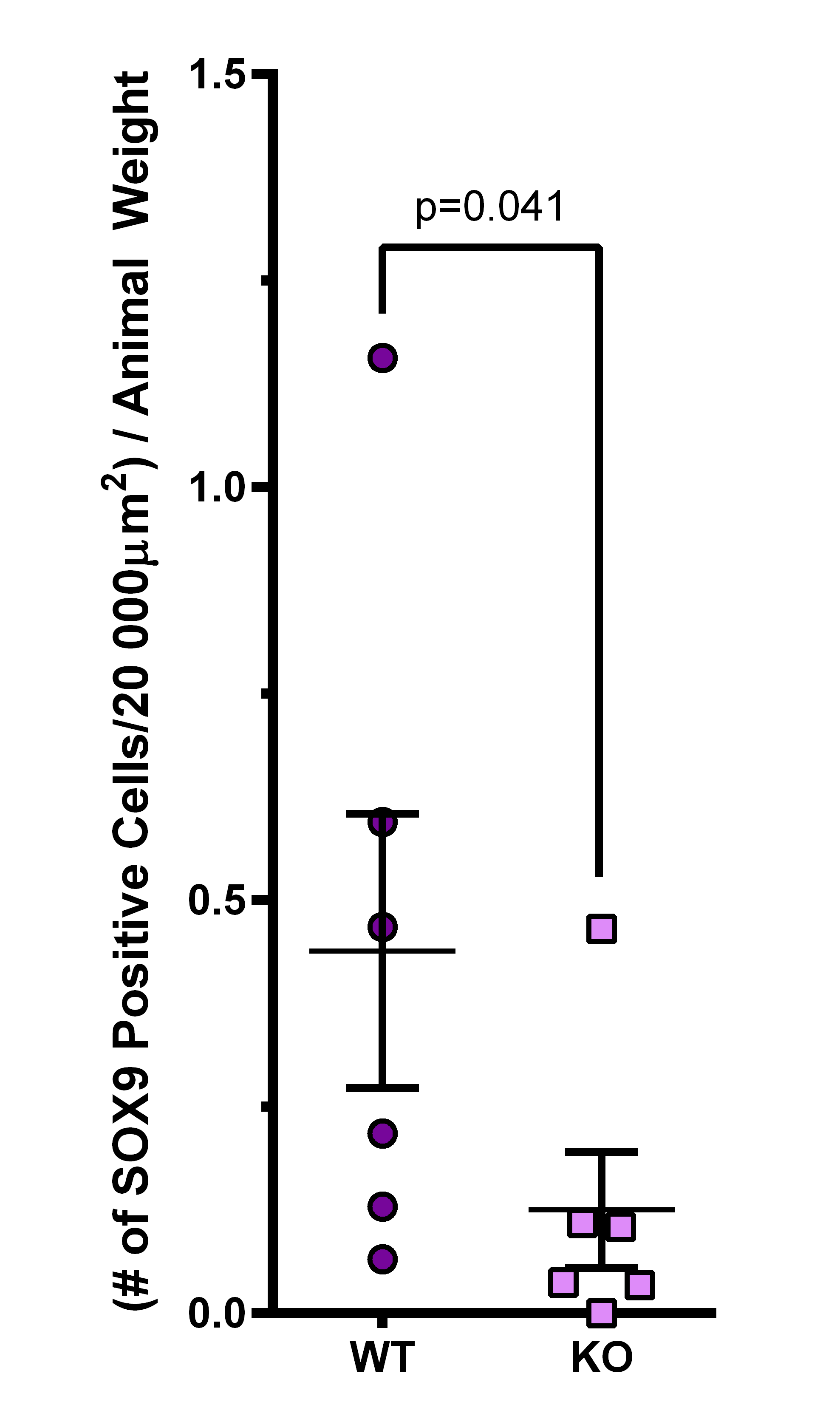

Supplement: S4 Fig — The number of SOX9 positive cells within a 200 x 100 μm box set at the articular cartilage surface of the lateral tibial plateau were counted and normalized to the animal’s weight to correct for any variability caused by differences in joint loading and animal size. Dusp1 KO mice show decreased numbers of SOX9 positive cells relative to WT controls. Data is presented as individual data points with mean ± SEM. Data analyzed using Mann-Whitney test. (TIF) [file pone.0142822.s004.tif]
