# Supplementary material for: Deletion of Dual Specificity Phosphatase 1 Does Not Predispose Mice to Increased Spontaneous Osteoarthritis
Source: PLoS One. 2015 Nov 12;10(11):e0142822. doi: 10.1371/journal.pone.0142822 (PMC4643037; doi:10.1371/journal.pone.0142822)
Supplement: S1 Table — DUSP1 WT (Dusp1 -/- ), Het (Dusp1 +/-), KO (Dusp1 -/-) animals live and healthy (Live) at the end of the experiment, or removed from the experiment due to recurrent skin ulcerations/died of unknown causes (Dead/Rem.). (DOCX) [file pone.0142822.s005.docx]

| **Female** | | | | **Male** | | | |
| --- | --- | --- | --- | --- | --- | --- | --- |
| **Genotype** | **Live** | **Dead/Rem.** | **% Live** | **Genotype** | **Live** | **Dead/Rem.** | **% Live** |
| WT | 9 | 1 | 90.0 | WT | 7 | 1 | 87.5 |
| Het | 8 | 1 | 88.9 | Het | 5 | 0 | 100.0 |
| KO | 9 | 2 | 81.8 | KO | 7 | 1 | 87.5 |
