# Supplementary material for: Deletion of Dual Specificity Phosphatase 1 Does Not Predispose Mice to Increased Spontaneous Osteoarthritis
Source: PLoS One. 2015 Nov 12;10(11):e0142822. doi: 10.1371/journal.pone.0142822 (PMC4643037; doi:10.1371/journal.pone.0142822)
Supplement: S2 Table — Articular cartilage damage was evaluated using the OARSI recommended scoring system. SEM, standard error of the mean; WT, wild type; KO, knockout; LTP, lateral tibial plateau; LFC, lateral femoral condyle; MTP, medial tibial plateau; MFC, medial femoral condyle. (DOCX) [file pone.0142822.s006.docx]

| **Sex** | **Genotype** | **Quadrant** | **Mean ± SEM** | **Median** | **Min** | **Max** |
| --- | --- | --- | --- | --- | --- | --- |
| Female | WT | LTP | 2.9 ± 0.5 | 2.8 | 1.2 | 4.5 |
|  |  | LFC | 0.6 ± 0.1 | 0.7 | 0.2 | 1.0 |
|  |  | MTP | 9.1 ± 1.9 | 7.8 | 4.2 | 20.7 |
|  |  | MFC | 1.5 ± 0.3 | 1.3 | 0.5 | 3.0 |
|  |  | Total Joint | 14.1 ± 2.1 | 12.8 | 7.7 | 26.7 |
|  | KO | LTP | 3.8 ± 0.6 | 4.0 | 1.7 | 7.2 |
|  |  | LFC | 1.1 ± 0.5 | 0.5 | 0.0 | 4.3 |
|  |  | MTP | 7.4 ± 1.0 | 6.9 | 4.0 | 12.8 |
|  |  | MFC | 1.2 ± 0.3 | 1.1 | 0.0 | 2.7 |
|  |  | Total Joint | 13.5 ± 1.2 | 12.9 | 8.8 | 19.0 |
| Male | WT | LTP | 3.8 ± 0.4 | 4.0 | 2.3 | 4.5 |
|  |  | LFC | 0.6 ± 0.3 | 0.5 | 0.0 | 1.5 |
|  |  | MTP | 7.7 ± 2.7 | 6.0 | 2.5 | 18.0 |
|  |  | MFC | 3.9 ± 1.7 | 2.3 | 0.5 | 10.0 |
|  |  | Total Joint | 15.9 ± 4.6 | 10.3 | 8.8 | 33.5 |
|  | KO | LTP | 3.0 ± 0.4 | 3.5 | 1.8 | 3.8 |
|  |  | LFC | 0.7 ± 0.4 | 0.5 | 0.0 | 2.0 |
|  |  | MTP | 8.2 ± 1.5 | 6.3 | 5.5 | 13.0 |
|  |  | MFC | 1.7 ± 0.7 | 1.0 | 0.3 | 4.0 |
|  |  | Total Joint | 13.4 ± 2.1 | 12.5 | 9.0 | 21.0 |
