# Supplementary material for: Deletion of Dual Specificity Phosphatase 1 Does Not Predispose Mice to Increased Spontaneous Osteoarthritis
Source: PLoS One. 2015 Nov 12;10(11):e0142822. doi: 10.1371/journal.pone.0142822 (PMC4643037; doi:10.1371/journal.pone.0142822)
Supplement: S3 Table — Articular cartilage thickness was measured from the cartilage surface to the subchondral bone. SEM, standard error of the mean; μm, micrometers; WT, wild type; KO, knockout; LTP, lateral tibial plateau; LFC, lateral femoral condyle; MTP, medial tibial plateau; MFC, medial femoral condyle. (DOCX) [file pone.0142822.s007.docx]

| **Sex** | **Genotype** | **Quadrant** | **Mean ± SEM (µm)** | **Median (µm)** | **Min (µm)** | **Max (µm)** |
| --- | --- | --- | --- | --- | --- | --- |
| Female | WT | LTP | 113.8 ± 3.3 | 113.4 | 101.9 | 131.7 |
|  |  | LFC | 90.6 ± 2.5 | 87.4 | 83.7 | 103.5 |
|  |  | MTP | 117.3 ± 4.4 | 119.0 | 101.4 | 137.4 |
|  |  | MFC | 92.0 ± 4.5 | 92.1 | 67.4 | 108.3 |
|  | KO | LTP | 113.7 ± 4.0 | 112.4 | 100.9 | 128.2 |
|  |  | LFC | 88.4 ± 3.0 | 86.4 | 80.0 | 104.0 |
|  |  | MTP | 120.3 ± 4.6 | 117.8 | 105.7 | 143.9 |
|  |  | MFC | 87.4 ± 4.9 | 83.8 | 71.3 | 116.1 |
| Male | WT | LTP | 115.3 ± 5.0 | 120.8 | 96.3 | 123.7 |
|  |  | LFC | 103.5 ± 3.6 | 106.4 | 94.6 | 113.4 |
|  |  | MTP | 131.3 ± 7.5 | 138.1 | 107.8 | 150.8 |
|  |  | MFC | 98.2 ± 6.7 | 91.3 | 85.4 | 119.7 |
|  | KO | LTP | 112.3 ± 4.8 | 110.8 | 96.3 | 123.2 |
|  |  | LFC | 104.3 ± 8.4 | 98.9 | 88.0 | 136.3 |
|  |  | MTP | 134.2 ± 3.7 | 131.3 | 127.8 | 148.5 |
|  |  | MFC | 99.0 ± 3.5 | 101.5 | 85.4 | 105.5 |
